# Protective Effects of Sulforaphane Preventing Inflammation and Oxidative Stress to Enhance Metabolic Health: A Narrative Review

**DOI:** 10.3390/nu17030428

**Published:** 2025-01-24

**Authors:** Inês Alves, Edilene Maria Queiroz Araújo, Louise T. Dalgaard, Sharda Singh, Elisabet Børsheim, Eugenia Carvalho

**Affiliations:** 1 Institute for Research and Innovation in Health (i3S), University of Porto, 4200-135 Porto, Portugal; inesa@ipatimup.pt; 2 Arkansas Children’s Research Institute, Little Rock, AR 72202, USA; eborsheim@uams.edu; 3 Nutritional Genomics and Metabolic Dysfunctions Research and Extension Center, Department of Life Sciences, State University of Bahia, Salvador 41195001, BA, Brazil; emaraujo@uneb.br; 4 Department of Science and Environment, Roskilde University, Universitetsvej 1, DK-4000 Roskilde, Denmark; ltd@ruc.dk; 5 Division of Hematology & Oncology, Department of Internal Medicine, Texas Tech University Medical Sciences Center, Lubbock, TX 79430, USA; sharda.singh@ttuhsc.edu; 6 Department of Pediatrics & Department of Geriatrics, University of Arkansas for Medical Sciences, Little Rock, AR 72202, USA; 7 Arkansas Children’s Nutrition Center, Little Rock, AR 72202, USA; 8 CNC-UC—Center for Neuroscience and Cell Biology, University of Coimbra, 3004-504 Coimbra, Portugal; 9 CIBB—Center for Innovative Biomedicine and Biotechnology, University of Coimbra, 3004-504 Coimbra, Portugal; 10 Institute for Interdisciplinar Research, University of Coimbra, 3030-789 Coimbra, Portugal

**Keywords:** sulforaphane, Nrf2, inflammation and oxidative stress, metabolic diseases, nutraceutical, metabolic syndrome, healthy aging

## Abstract

The worldwide obesity epidemic has led to a drastic increase in diabetes and cardiovascular disease in younger generations. Further, maintaining metabolic health during aging is frequently a challenge due to poor diets and decreased mobility. In this setting, bioactive nutrients that are naturally occurring antioxidants, such as sulforaphane (SFN), are of high nutritional interest. SFN, a bioactive compound that is present in cruciferous vegetables, is a molecule that protects cells from cytotoxic damage and mitigates oxidative stress, protecting against disease. It exerts its action through the activation of the transcription factor nuclear factor erythroid 2-related factor 2 (Nrf2). Many studies have been performed in animals and humans to evaluate its effects on cancer, brain health, and neurodegenerative disorders. However, fewer clinical studies have been performed to evaluate its effects on insulin resistance and the development of type 2 diabetes mellitus (T2DM) across the lifespan. Given that, in some parts of the world, particularly in Europe, the population is growing older at a significant rate, it is crucial to promote healthy habits (healthy foods, dietary pattern, precision nutrition, and physical activity) from an early stage in life and across the lifespan to avoid debilitating health conditions occurring during adulthood and aging. Thus, in this narrative review, we discuss the protective effects of SFN supplementation on inflammatory and oxidative stress pathways and relate them to metabolic disease.

## 1. Introduction

One-fifth of the European population is above 65 years of age [1]. Soon, the number of older adults will exceed that of children, with an extraordinary rise in the number of people reaching extreme old age [2]. Considering both the proportion of older people and the expected increase in lifespan worldwide, it will be a challenge to keep the aging population in good health, with a sustained sense of well-being and extended periods of social engagement and productivity; these criteria are currently all met in around 14.7% of this population [2]. A major concern is that the older generation will be burdened with higher rates of illness, disability, and dependency. The increase in the proportion of older people is mainly due to the unprecedented medical and research advances over the last few decades, but also due to the decrease in birth rates in developed countries. Despite these medical advances, aging may soon represent an economic and social burden for society if it is accompanied by unhealthy lifestyles, such as inactivity, unhealthy habits, and the intake of poor-quality diets [3,4,5,6]. Optimal nutrition and physical activity throughout the lifespan are essential for healthier and higher-quality life [7].

Aging is generally considered irreversible due to the accumulation of molecular and cellular damage within an organism’s somatic cells and tissues. Cellular aging is associated with the progressive loss of compensatory protective mechanisms and/or the degradation of cellular functions, ultimately resulting in impaired homeostasis. Factors responsible for the loss of cellular homeostasis include genomic instability, telomere attrition, epigenetic alterations, loss of proteostasis, deregulated nutrient sensing, mitochondrial dysfunction, cellular senescence, stem cell exhaustion, and altered intercellular communication [8]. Oxidative stress and related cellular damage are primary factors that contribute to multiple health problems including sarcopenia, cognitive dysfunction, and cardiovascular disease. Several chemically and functionally diverse scavengers of reactive oxygen species and lipid peroxidation have been experimentally evaluated for their ability to mitigate oxidative stress, but with limited success [9,10,11]. The major reasons for the lack of benefits obtained from antioxidants may be due to their low bioavailability and/or low scavenging efficacy against oxidants and electrophiles in cellular systems, as well as potential secondary reactions with other biomolecules [12].

Sulforaphane (SFN) is a non-toxic phytochemical compound found in cruciferous vegetables, such as broccoli, Brussels sprouts, and cauliflower, with an absolute bioavailability of around 80% [13]. It has been identified as a promising nutraceutical given its ability to induce the expression of several endogenous antioxidant enzymes, such as glutathione S-transferase (GST), NAD(P)H:quinone oxidoreductase-1 (NQO1), hemeoxygenase-1 (HO-1), and glutamate–cysteine ligase (GCL) via the activation of the transcription factor nuclear factor-erythroid-2-related factor 2 (Nrf2 encoded by the NFE2L2) gene [14]. Moreover, accumulating evidence is showing that SFN can also inhibit Toll-like-receptor oligomerization, and the consequent Nf-kb activation and Th1/Th17 polarization, meaning that it is also an important player in the inflammatory regulatory mechanisms. Although it is considered non-toxic at typical food consumption doses, it should be administered with caution, when taking into consideration higher doses. Previous studies have reported toxicity occurring at higher doses of sulforaphane in rats, which underscores the need for careful attention to the risk–benefit analyses, as well as the determination of the therapeutic or prophylactic doses [15]. It was noted that high doses of SFN injected intraperitoneally into rats led to significant side effects, including marked sedation (at 150–300 mg/kg), hypothermia (at 150–300 mg/kg), disturbed motor coordination (at 200–300 mg/kg), decreased skeletal muscle strength (at 250–300 mg/kg), and death (at 200–300 mg/kg). These findings highlight the need for responsible dosing strategies. Thus, the authors suggested that in long-term preventive interventions with SFN or broccoli-based formulations, it is essential to consider the dose, timing, and duration carefully.

SFN is generated from the precursor isothiocyanate glucoraphanin followed by digestion with the enzyme myrosinase in the intestine. Due to its potent antioxidant and anti-inflammatory properties, SFN has gained attention as a therapeutic nutraceutical for cancer, autoimmunity, infection, and, most recently, metabolic diseases [16]. A more successful strategy for protecting against oxidative and electrophilic injury during aging may be the induction of endogenous antioxidants and phase 2 enzymes, which could have tremendous potential for improving the quality of life and lifespan in the aging population [17]. Besides activating Nrf2, SFN also protects mitochondrial function during oxidative stress [18,19,20], which represents another advantage over conventional antioxidants.

SFN’s high bioavailability profile is one of its advantages. In people in good general health, oral administration of 200 µmol broccoli sprout isothiocyanates resulted in a peak of 0.943–2.27 µmol/L of isothiocyanate in plasma one hour after ingestion [21,22,23]. SFN is eventually degraded in the liver, with studies showing a half-life of 1.7 ± 0.13 h and a final clearance rate of 369 ± 53 mL/min. At 8 h, cumulative excretion was more than 50% of the ingested dose [21,22,23].

In this review, we discuss how metabolic and immune pathways can be programmed through a nutraceutical approach, specifically by SFN supplementation in the diet. The emerging field of geroscience, intersecting basic aging biology, chronic diseases, and health, to elucidate molecular mechanisms of aging and age-associated chronic diseases, has identified specific pathways, including oxidative stress, as important targets for enhancing the quality of life in older people [24]. Interestingly, some of these pathways, namely mitochondrial efficiency and accumulation of reactive oxygen species (ROS), can be modulated by precision nutrition, including SFN [25]. 

## 2. Age-Associated Loss of the Nrf2 Oxidative Stress Response

**Figure 1:**
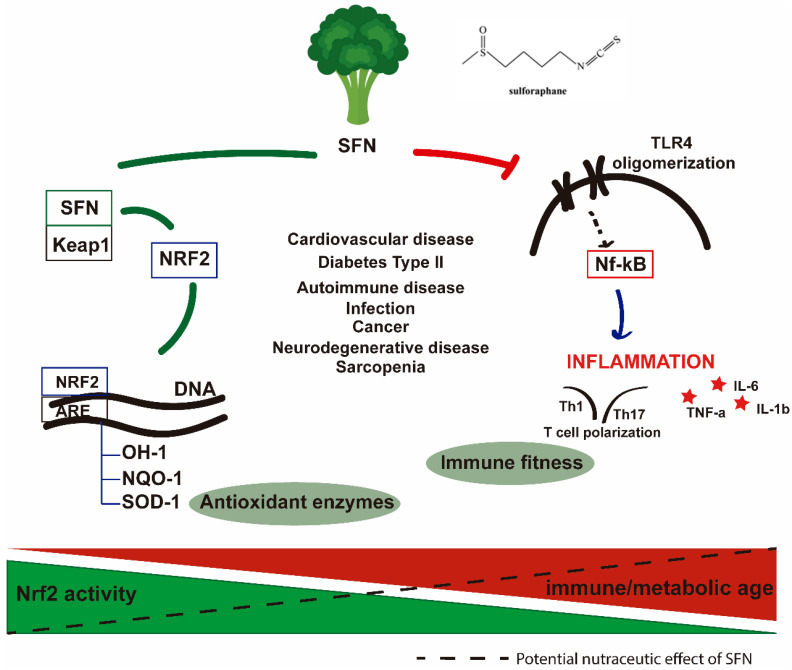
The umbrella protective effect of SFN in immune/metabolic aging. During the aging process, Nrf2 activity decreases, resulting in an inefficient cellular stress response. SFN acts on this axis by the direct induction of Nrf2-ARE binding. Simultaneously, inflammation is also a hallmark of aging. Levels of inflammation are increased in the elderly, and this is associated with a poor quality of the aging process. The action of SFN relies also on the inhibition of TLR4 oligomerization resulting in a decreased activation of the NF-kB pathway and the pro-inflammatory profile of the cell. SFN: sulforaphane; Nrf2: nuclear factor-erythroid factor 2-related factor 2; Keap1: Kelch-like ECH-associated protein 1; ARE: antioxidant response element; OH-1: Heme oxygenase-**1**; NQO-1: NADPH quinone oxidoreductase enzyme; SOD-1: superoxide dismutase; TLR4: Toll-like receptor; Nf-κB: nuclear factor kappa-light-chain-enhancer of activated B cells; TNF-a: tumor necrosis factor; IL-6: interleukin 6; IL-1b: interleukin-1b.

Under homeostatic conditions, the transcription factor Nrf2 is sequestered in the Kelch-like ECH-associated protein 1 (Keap-1) complex in the cytosol (Figure 1). This complex is disrupted when the cell senses stressful events, including increased ROS levels, allowing Nrf2 to translocate to the nucleus. Once in the nucleus, Nrf2 binds to the antioxidant response element (ARE) in the promoter region of antioxidant, pro-survival, anti-inflammatory, and damage-repair genes [26,27]. The Keap1-Nrf2-ARE pathway is responsible for the expression of genes involved in the protective response against cellular stress, including antioxidant enzymes such as GST, NQO1, HO-1, and GCL. SFN is also important for the activation of genes involved in autophagy and proteasomal degradation [14]. Furthermore, Nrf2 can also bind to the promoter of key enzymes, such as 8-Oxoguanine DNA glycosylase, in the base excision repair pathway, revealing its close association with specific DNA repair mechanisms [28].

Cellular responses to stress rely on the Keap1-Nrf2-ARE axis, and when its activity is impaired, deleterious toxic compounds may be trapped within the cell. This has been observed in Nrf2^−/−^ mice, which present increased susceptibility to oxidative stress damage, such as butylated hydroxytoluene-induced acute pulmonary injury [29], acetaminophen-induced liver toxicity [30], and hyperoxia-induced lung injury. Moreover, age-associated cellular and organ damage has been associated with the impaired activity of Nrf2, including in chondrocytes [31], cardiomyocytes [32], the neuronal system [33], skeletal muscle and the kidney [34], and the liver [35]. The decrease in Nrf2 activity culminates in the downregulation of antioxidant enzymes necessary for a cellular stress response balance [36]. Adding to this impairment in protective responses, mitochondrial efficiency tends to also decrease with age [18]. This is, in part, through the acquisition of mutations in mitochondrial DNA, which may lead to the accumulation of abnormal respiratory chain proteins, causing partial uncoupling and further accumulation of ROS [37]. Interestingly, overexpression of Nrf2 is cytoprotective in several tissue injuries [38,39,40,41]. These data demonstrate that Nrf2 dysregulation with age may be a pivotal player in the loss of the cellular redox status.

Decreased Nrf2-ARE binding activity with aging may potentiate the phenotypes observed in genetically modified mouse models. However, less is known about the mechanisms that directly impair the Nrf2-ARE activity or cause its decreased expression levels with age. Studies have shown that Nrf2 ablation increases susceptibility to several age-associated diseases. For example, the absence of Nrf2 in an Alzheimer experimental mouse model led to increased levels of Amyloid-beta precursor protein (APP), and beta-amyloid plaque formation increased faster in the mice depleted of Nrf2 [42,43]. In addition, high levels of oxidative stress damage were observed in the spinal cord of a multiple sclerosis (MS)-like mouse model [44]. These findings were accompanied by mitochondrial dysfunction in the brains of these mice, such as decreased levels of glutathione and increased activity levels of complex II and III with no significant differences in ATP levels [45]. The close link between MS and mitochondrial dysfunction suggests the Nrf2-ARE pathway is an important mediator of the pathogenesis [46]. The loss of the Nrf2 gene in this MS-like mouse model was paralleled with an increased susceptibility to the disease and with the severity of the disease course. These alterations were accompanied by exacerbated pro-inflammatory cytokine levels, spinal cord damage, and axonal degeneration [46,47]. Furthermore, oxidative stress, mitochondrial dysfunction, and neuroinflammation were identified as important contributors to the development and progression of Parkinson’s disease (PD) [46,48]. In an experimental mouse model of PD, activity levels of Nrf2 were decreased, while ablation of the *Nfe2l2* gene (encoding Nrf2) in these mice led to further exacerbation of the PD clinical course. In human studies, Nrf2 expression and activity were also found to be decreased in nigral dopaminergic neurons in PD patients [49]. Huntington’s disease (HD), another neurodegenerative disease, has also been associated with high levels of peripheral oxidative stress markers, including plasma lipid peroxidation, protein and mtDNA oxidative damage, and low levels of glutathione [46,50]. As a proof of concept, in the 3-nitroproprionic acid (NP)-induced HD mouse model, Nrf2 activation was decreased, and genetic ablation of Nrf2 in these mice led to increased HD susceptibility. Conversely, chemical induction of Nrf2 activity was sufficient to protect animals from 3-NP-induced HD [51].

Ischemic stroke, another biological process associated with impaired neurological function, implicates a peak of oxidative stress during the reperfusion phase. Mouse models have demonstrated that Nrf2 ablation has led to enhanced inflammatory responses and cognitive impairment after a brain ischemia event [46,52]. Nrf2-impaired activity has also been observed in other age-associated disorders, such as macular degeneration [53], sarcopenia [54,55], and bone frailty [56].

Given the key role of Nrf2 in the response to oxidative stress, it is expected that lower Nrf2 activity in metabolism-associated disorders would have a significant negative impact during aging [46]. However, the relationship between ROS and lifespan is complex. ROS can have both beneficial and detrimental effects on longevity depending on species and conditions [17]. In fact, obesity and associated excess calorie intake potentiate and drive low-grade chronic inflammation and an imbalance in the redox status [57,58]. Caloric restriction is one of the highlighted strategies for improving longevity and decreasing inflammation [59]. This is due to both the increased autophagy occurring in highly metabolically active cells and an increase in the Nrf2 expression and activity, resulting in enhanced cellular protection [60]. However, westernized diets, rich in lipids and simple sugars, are known to promote chronic low-grade inflammation and potentially contribute to the development of metabolic disorders, such as insulin resistance and T2DM. Of interest, high-fat-fed mice with cell-specific deletion of Nrf2 in adipocytes displayed decreased glucose tolerance, but when Nrf2 deletion occurred in hepatocytes, mice presented a trend toward improved insulin sensitivity [61].

## 3. Nrf2 Bridging the Link Between Inflammation and Oxidative Stress

During aging, inflammation levels increase, a phenomenon known as inflammaging. This is closely linked with the efficient activation of the cellular redox response through Nrf2 function [62]. Inflammation occurs in all living tissues with different degrees of severity, contributing to the accumulation of ROS and cellular damage [17,63]. Conversely, the accumulation of ROS has also been identified as a key trigger of immune responses [17,62]. Therefore, this close association between oxidative stress and inflammation again brings the Nrf2 activity into the discussion [62,63]. The interplay between the cellular redox status and inflammation can be explained by signaling interactions between Nrf2 and the master immune and inflammatory regulator nuclear factor kappa-light-chain-enhancer of activated B cells (NF-κB). For example, NF-κB can directly inhibit Nrf2-ARE signaling [62,63], but Nrf2 can also negatively regulate NF-kB signaling through the transcription of antioxidant genes [64]. In a mouse model of autoimmunity, in scurfy mice ablated for regulatory T cells, Nrf2 systemic activation (*Keap1* deletion) led to a decrease in T-cell activation and in turn a suppression of pro-inflammatory cytokines, resulting in the amelioration of tissue inflammation [65]. Interestingly, genes associated with the Th17 response, usually a major axis of autoimmune responses, such as *Il17a* and *Rorc*, display several ARE repressor motifs, enabling Nrf2 inhibition of IL-17-mediated immune responses [66].

Some studies have implicated Nrf2 activation in the development of immune-related diseases. In the context of autoimmune disease, Nrf2 ablation resulted in increased severity of colitis in a dextran sulfate sodium (DSS)-induced mouse model [67]. In addition, aged female Nrf2^−/−^ mice naturally developed a severe form of glomerulonephritis [68].

## 4. SFN in Age-Related Disorders—A Nutraceutical in Healthy Aging

Due to the specific inefficiency of the cellular stress response in aging, specifically through impaired Nrf2 activity and not necessarily through its protein expression, several Nrf2 activity inducers have been tested in the human disease context. Physical exercise, SFN supplementation, calorie restriction (including fasting), and ingestion of natural compounds such as quercetin, melatonin, vitamin E, luteolin, and alpha lipoic acid are some of the most potent Nrf2 activators, presenting the highest increase in Nrf2-ARE binding activity [32,69,70]. Therefore, the nutraceutical potential of SFN has drawn attention, especially in the context of healthy aging. Studies of long-lived species have demonstrated higher Nrf2 activity in species with longer lifespan; a 10-year increase in lifespan was associated with a 1.4-fold increase in Nrf2 activity, whereas its protein level was less crucial [71].

Attention to potential therapeutic targets of Nrf2-dependent inflammation and redox status has increased during the last decade. Given that the Nrf2 activity deceases with age, it is crucial to define novel strategies for potentiating the Nrf2 axis. Several Nrf2 inducers have been studied, including phytochemicals (e.g., quercetin, luteolin), alpha lipoic acid, physical exercise, and caloric restriction. Physical exercise is, undoubtedly, one of the most accepted and efficient strategies for improving quality of life and delaying the aging process [72]. In a recent nematode study, the authors indicate that SFN prolongs the life and health span of C. elegans through insulin/IGF-1 signaling. Moreover, oral supplementation of SFN has been shown to dramatically prevent skin aging, through the activation of the Keap1-Nrf2 pathway [73]. These results provide the basis for a nutritional SFN-enriched strategy for the promotion of healthy aging and disease prevention [74]. As a result, precision nutrition using phytochemicals has become an attractive therapeutic possibility for metabolic and inflammatory diseases across the lifespan. In fact, several phytochemicals have been tested in aging-associated disorders, including SFN, which has been characterized as having high bioavailability with its blood concentration peaking around 1 h after ingestion [22]. This is due to its lipophilicity and is in contrast to other phytochemicals tested for Nrf2 activation. Together with SFN’s ability to induce gene transcription of protective pathways, these characteristics reveal the strong nutraceutical potential of this natural bioactive compound [22,62].

## 5. SFN in Cardiometabolic Diseases

Cardiovascular and metabolic diseases are major causes of morbidity and mortality not only in the aging population, but lately also at younger ages [75,76]. This is partly due to a shift in dietary habits in recent decades [4], which has led to a massive rise in the incidence of metabolic syndrome and cardiometabolic risk factors. Metabolic syndrome is a cluster of the most dangerous heart attack risk factors: diabetes and elevated fasting plasma glucose, abdominal obesity, high cholesterol, and high blood pressure [77]. According to the International Diabetes Federation (IDF) definition [75], for a person to be defined as having metabolic syndrome, they must have central obesity (defined as waist circumference with ethnicity-specific values) plus any two of the following four factors: raised plasma triglycerides, reduced plasma HDL cholesterol, raised blood pressure, raised fasting plasma glucose, or specific treatment for elevated circulating lipids. The risk for these co-morbidities increases as the age of T2DM onset decreases, potentially leading to decreased lifespan [78,79,80]. Interestingly, this phenomenon is occurring not only in Western societies, but also in places like India, where the population is largely vegetarian, and Africa where people feed mainly on rice. In India, people rely mostly on cereals for their diet, and the obesity incidence is not as high as in Western countries. Despite this, the prevalence of metabolic diseases, including T2DM, is increasing rapidly [81,82,83]. Thus, many in the Indian population are considered “metabolically obese”, since metabolic syndrome occurs despite weight being normal.

A common feature of these diseases is the high levels of oxidative stress, mitochondrial dysfunction, and inflammation, most likely due to poor nutritional habits [62]. SFN-dependent Nrf2 activation is an efficient tool for restoring cardiometabolic homeostasis and preventing cellular damage. SFN was able to inhibit the NF-kB DNA binding activity and downregulate TNF-α-mediated induction of intercellular adhesion molecule 1 (ICAM-1) in endothelial cells, suppressing inflammation in atherosclerotic lesions in preclinical models [84,85,86]. Moreover, SFN pre-treatment prevented oxidized (ox) LDL-induced ROS production, NF-kB nuclear translocation, ICAM, vascular cell adhesion protein 1 (VCAM), and E-Selectin expression, as well as monocyte adhesion to endothelial cells, in an in vitro model [84]. Furthermore, in a rabbit model of hypercholesterolemia, animals supplemented with SFN in the diet for 4 weeks were protected against elevation of total cholesterol, LDL-C, CRP, and LDH, which was accompanied by a marked decrease in NF-kB in the aortic tissue [87]. SFN showed an efficient inhibition of platelet aggregation and thrombus formation, through the inhibition of PI3K/Akt pathway, in a thrombotic model, both in vivo (mice) and in vitro (human platelets) [88,89]. These studies point to the relevant value of SFN supplementation in the diet as a strategy for decreasing metabolic impairment. SFN’s significant reduction in oxidative stress and inflammation-associated pathologies has led to a significant improvement in the quality of life across the lifespan [90]. In fact, a phase 1 study showed that SFN supplementation for one week led to improved cholesterol metabolism (total cholesterol and LDL cholesterol decreased and HDL cholesterol increased significantly) and decreased oxidative stress markers (reduced plasma phosphatidylcholine hydroperoxide, 8-isoprostane and 8-OHdG and increased CoQ_10_H_2_/CoQ_10_ ratio) in 12 healthy subjects [91].

More recently, SFN interventions against metabolic syndrome, namely in the context of impaired glucose response and T2DM, as well as hypertension and vascular disease, have proven valuable [92,93]. Despite the apparent link between the antioxidant response and protection against metabolic syndrome, the SFN intervention in this context is still unclear, and the studies are scarce. One of the first human studies was performed in 2011; in it, 81 patients with T2DM were treated with 5 and 10 g/day of broccoli sprout powder for four weeks. Patients treated with SFN showed significantly decreased levels of malondialdehyde and ox-LDL, along with reduced oxidative stress index [94]. The authors later demonstrated that the same supplementation set-up induced a significant reduction in the serum insulin concentration, as well as the HOMA-IR index [95]. In a recent animal study evaluating the use of SFN in weight loss, the authors concluded that its anti-obesity effect required functional signaling of the leptin receptor. In addition, the results suggested that skeletal muscle was the most notable site of action of SFN, whose peripheral action of Nrf2 signals alleviates leptin resistance and suppresses fatty acid synthesis, leading to protection against obesity. The results suggested clinical evaluation of SFN for weight loss and metabolic disorders associated with obesity [96].

In another study, mice on a high-fat diet (HFD) were supplemented with SFN (100 µmol/kg for six weeks); this supplementation induced a marked increase in the insulin receptor substrate 1 (IRS-1), as well as enhanced GLUT4 translocations to the muscle cell membrane [97]. In another rodent study where T2DM was induced by HFD and streptozotocin, SFN treatment (2 and 10 mg/kg for 8 weeks) resulted in increased serum insulin levels, enhanced HOMA-β index, and decreased fasting glucose [98]. These studies nurture the need for more extensive clinical studies and detailed analysis of Nrf2 and SFN-mediated protection of pre-diabetic and already-established diabetic subjects.

In highly energetic tissues, such as skeletal and cardiac muscle, ROS and reactive nitrogen species (RNS) levels are sufficiently elevated to induce massive cellular damage if cytoprotective mechanisms are impaired. In fact, 12 weeks of SFN supplementation in free-of-disease mice has shown healthy mitochondrial function, cardiac function, exercise capacity, glucose tolerance, and activation/differentiation of skeletal muscle satellite cells during aging [17]. This evasion of aging-induced mitochondrial dysfunction, sarcopenia, and cardiac impairment was associated with restored Nrf2-ARE binding levels, which resulted in increased redox protection in the skeletal and cardiac muscle tissues [18].

During aging, sarcopenia or the loss of skeletal muscle mass can be associated with a gain in adipose tissue [99]. This is generally associated with lower dietary protein intake and a sedentary lifestyle, but is also due to intrinsic aging-associated mechanisms, such as myosteatosis [100], resulting from several factors, including estrogen deficiency, glucocorticoid treatments, and disuse atrophy. In a study targeting obesity, mice were fed a high-fat diet and supplemented with SFN for 6 weeks; the results displayed attenuated HFD-induced visceral adiposity, adipocyte hypertrophy, and fat accumulation in the liver. Moreover, blood levels of total cholesterol and leptin were lower in SFN-supplemented mice. The mechanisms behind the prevention of the obesity-associated phenotype rely on the decreased expression of PPARγ, C/EBPα levels, and increased levels of adiponectin, mediated by AMPK activation [22,62].

## 6. SFN in Immune Fitness

Oxidative stress is often related to immune dysfunction which is enhanced in aging. A balanced and effective immune response is crucial for maintaining homeostasis in the host either by resolving infections, avoiding autoimmune reactions, or hampering the growth of malignant cells. Impaired innate immunity directly contributes to the tumor immune escape due to deficient immune surveillance or an uncontrolled immune response resulting in chronic inflammation [101]. These processes are explained by the loss of immune fitness which relies on the ability of the immune network to respond to inflammatory stimuli and mount a proper response, but also by its ability to silence the pro-inflammatory response and resolve the inflammation. Given the relevance of Nrf2 in the activation or suppression of specific and important immune-mediated pathways, the activation of Nrf2 through SFN supplementation seems to be of high relevance. SFN exerts a pleiotropic effect on immunological responses by the activation of Nrf2, which triggers cellular defense mechanisms. There is induction of Phase II detoxifying enzymes as well as antioxidant enzymes and downregulation of Phase I enzymes by inactivation of NFκβ. The final effect of SFN varies with cell type [102].

Overweight in the absence of comorbidity is a good model in which to study basal chronic inflammation. In a clinical trial with healthy overweight subjects (24.9–29.9 kg/m^2^, with no other diagnosed diseases), IL-6 and CRP were used to monitor inflammation during fresh broccoli sprout supplementation (30 g/day of broccoli sprouts for 10 weeks) [103]. Interestingly, the authors observed a decrease in plasma IL-6 and CRP protein levels associated with increased SFN levels in the urine. After the intervention period, a prolonged decrease in IL-6 was found, whereas CRP returned to basal levels after 90 days of follow-up. Moreover, SFN supplementation also resulted in the downregulation of basal IL-6 and CRP protein levels in T2DM patients [104]. The mechanistic pathway behind SFN-mediated control of the innate immune response in metabolic homeostasis is still to be elucidated. Specifically, for Toll-like receptor 4 (TLR4)-mediated inflammation, it was shown that SFN was able to suppress oligomerization of this receptor through physical constraint in a thiol-dependent manner [105].

The direct regulation of TLR4 by SFN may have a strong impact on immune responses, given that oligomerization of TLR4 is an important driver in several immune-mediated diseases. For example, in an experimental model of autoimmune encephalomyelitis (EAE), the CD4^+^T helper (Th) type 1 and the Th type 17 immune responses are the major contributors to disease development [106]. The authors showed that 0.003% of SFN in food for 18 days after induction of the disease had a great impact on the protection of mice against EAE development. Th1 and Th17 polarization rely on levels of IL-12 and IL-23 from dendritic cells (DCs), and the suppression of DC TLR4 by SFN may be responsible for the significant reduction in Th1 and Th17 polarization. In addition, SFN was able to induce the expression of HO1, which, interestingly, was a result of HO1 binding to NF-kB p65, inhibiting the transcription activity at the IL-23a and IL-12b promoters [106]. Furthermore, a lupus-prone MRL^+/+^ mouse model of trichloroethene (TCE)-mediated autoimmunity, which is associated with the development of Systemic Lupus Erythematosus (SLE), systemic sclerosis (SS), and autoimmune hepatitis, was treated for 6 weeks with SFN. Splenocytes from these mice presented lower levels of oxidative stress and in turn were protected from environmental insults, with lower levels of IL-6, TNF-α, and IFNγ. Since TCE induces an artificial TLR4 oligomerization, this study agrees with the data previously described showing that SFN is able to inhibit TLR4 oligomerization, restricting NF-kB p65 activity [107]. Importantly, several environmental factors can act as severe inflammatory triggers, including, noise, air pollution, pesticides, food chemicals, dust, and tobacco usage. These pollutants can disrupt the sinonasal epithelial barrier, leading to an increase in the basal levels of inflammation at this site. On the other hand, in vitro SFN supplementation of human sinonasal epithelial cells demonstrated protective effect in the integrity of the epithelial barrier [108,109,110,111].

Another instance where SFN can be protective is in inflammatory bowel disease (IBD). IBD is characterized by an imbalanced inflammatory response in the gut. Gut leakage is another marker of aging, in which the epithelial gut barrier and immune defenses are impaired [112]. This is turn, allows for the entrance of pathogens into the intestinal lumen, exacerbating the immune response [113]. This chronic inflammation has also been associated with loss of redox protection, which leads to immune dysfunction and significant gut damage. In a mouse model of DSS-induced colitis, 2 weeks of supplementation with SFN (20 mg·kg^−1^·day^−1^) was shown to be protective, resulting in increased body weight, longer colon length, and reduced disease activity index [114]. This SFN-induced protection resulted in the recovery from DSS-induced dysbiosis [114]. Moreover, in a mouse model of bladder cancer, pre-treatment with 2.5 and 10 mg·kg^−1^·day^−^1 SFN also protected mice from developing tumors, and this was associated with the regulation of microbiota [115].

Furthermore, regarding host–pathogen responses, *H. pylori*-associated gastritis seems to be suppressed by SFN supplementation, also mediated by the activation of Nrf2. Fresh broccoli sprouts were taken daily by 48 infected subjects, and inflammatory markers such as IL-1β and TNF-α were decreased after 2 months of SFN supplementation [116,117]. Similarly, in a model of *S. aureus* infection, in vitro pre-treatment of infected mouse macrophages with SFN showed the suppression of pro-inflammatory genes, such as IL-1β, IL-6, TNF-α, and M1 markers, including CCR7, IL-23, and iNOS. The effect of SFN on the inhibition of *S. aureus*-mediated inflammation was observed to occur through the SFN-mediated inhibition of p38 and JNK phosphorylation [118]. The antimicrobial properties of SFN also encompass the inhibition of mycoplasmal lipopeptide-induced pulmonary infection in mice, through the inhibition of NF-kB as well as monocyte-derived pro-inflammatory cytokines [119]. The constitution and diversity of the microbiome have been demonstrated as crucial for systemic homeostasis. However, the aging population presents a less diverse microbiome, as well as increased levels of pathogenic strains that may contribute to the impaired immune system and the imbalance in metabolic homeostasis [115]. Interestingly, SFN supplementation of aging mice restored a healthy gut microbiome, similar to that observed in young mice, when compared with old non-treated mice [120]. This equilibrium of host–pathogen barrier in the intestine not only impacts gut homeostasis, but also has an effect in distant organs, mainly due to the loss of systemic immune fitness in homeostasis. For example, in a mouse model of bladder cancer, SFN treatment resulted in fewer submucosal capillaries, which was associated with a normalization of the microbiome dysbiosis and decreased IL-6 and IgA levels [115,121].

Importantly, in the context of the COVID-19 viral pandemic, the vulnerability of older people to succumbing to the SARS-CoV-2 infection was a major global issue [122]. Due to the associated metabolic comorbidities and/or age-associated loss of immune fitness, the aging population presented the most severe consequences of COVID-19 viral infection. This was in part due to the development of an exacerbated pro-inflammatory response, such as the well-known “cytokine storm” [123]. Indeed, authors have hypothesized a protective effect of Nrf2 activation, through SFN supplementation, against COVID-19 tissue damage and disease severity [124]. SFN acts by inhibiting the activation of the Nucleotide-binding domain leucine-rich repeat-containing family pyrin domain-containing 3 (NLRP3) inflammasome [125].

Moreover, in the context of cancer, prostate cancer, lymphoma, and colorectal cancer have an increased incidence in the aging population [126], either due to impaired immune response which allows the tumor’s immune escape or through the accumulation of mutations that impair cell cycle arrest [127]. Preliminary studies have demonstrated a potential role of SFN in the protection of prostate [128], colorectal [129], and breast cancer [130]. Moreover, current oncological therapies are highly cytotoxic, and adjuvant supplementation with SFN seems to protect against tissue damage promoted by these drugs [131,132,133]. Another study demonstrated the efficacy of cruciferous vegetables, enriched with SFN, in preventing breast cancer recurrence [134]. The evidence suggests that SFN supplementation could potentially be associated with current therapies, not only to increase therapeutic efficacy but also to diminish the cytotoxic effects [90].

## 7. SFN in Obesity and Metabolic Syndrome

SFN plays a crucial role in obesity, and therefore, this topic has already been extensively reviewed by others. Among its significant effects, it mitigates high-fat-diet-induced obesity by enhancing mitochondrial biogenesis in muscle [135]. Further, one of the key protective effects of SFN in white fat is its ability to induce browning [136]. More recently, SFN was shown to reduce obesity by reversing leptin resistance [97]. In contrast, much less is known about the effects of SNF in metabolic syndrome. Given that it plays a role in muscle and fat to alleviate obesity, it directly impacts diabetes and cardiometabolic syndrome [137]. However, further clinical studies are needed to establish the benefit of SFN in type 2 diabetes.

## 8. SFN Supplementation in Clinical Trials

Most SFN supplementation studies have been carried out in mouse models (Table 1), which raises the question about its translational relevant effect in human clinical trials. Several clinical trials have demonstrated that SFN positively contributes to metabolic protection in humans, either under homeostasis or in already established diseases, as shown in Table 2. In overweight individuals, broccoli sprouts lower inflammation markers, such as IL6 and CRP [103]. Broccoli sprout extracts also ameliorated fatty liver disease, through the down-modulation of the ALAT enzyme, as well as gamma-glutamyl transferase (GGT) and 8-hydroxydeoxyguanosine (8-OHdG) [138]. In subjects with T2DM, it was observed that broccoli sprouts decreased oxidative stress, with lower levels of oxidized LDL cholesterol [139], as well as decreased fasting glucose and HOMA-IR [95]. Other ongoing clinical trials highlight the importance of studying the in vivo impact of SFN in other metabolic and age-associated conditions, such as NAFLD, insulin resistance, T2DM, increased blood pressure, impaired fasting glucose, obesity and related chronic inflammation, skin aging, skin response to UV radiation, and doxorubicin-associated cardiac dysfunction, as described in Table 3.

## 9. Preventive Measures to Include SFN in Our Daily Diet—Treatment or Preventive Usage?

The collected studies on SFN interventions have shown beneficial effects when tissue and cellular homeostasis is lost. This has particular advantages in a population that reaches a longer lifespan, which is more susceptible to natural loss of homeostasis [142]. However, SFN interventions should be implemented as prophylactic measures to achieve the desired “healthy aging”. Fruit and vegetable consumption has increased over the years; however, the intake of cruciferous vegetables, rich in SFN, is still low [143,144]. Despite its apparent innocuity, SFN has a pleiotropic effect along diverse organs and conditions, which raises the question about the therapeutic concentration to use as a prophylactic intervention [145]. Therefore, the implementation of fresh SFN-rich food in the daily diet of healthy or elderly populations is an important precision medicine strategy for curbing oxidative stress and chronic inflammation. In a debilitated population, with associated diseases or syndromes, the concentration of SFN in the diet could also be increased by the promotion of the consumption of broccoli sprouts, which are much richer in SFN than the normally harvested broccoli.

The WHO action framework for developing and implementing public food procurement and service policies for a healthy diet has recommended the increase in consumption of whole grains, vegetables, fruit, nuts, and pulses, as well as the decrease in consumption of sodium and salt, sugars and fats, particularly trans fats [146]. Regarding these guidelines, some of the measures taken by European governments in public settings are as follows: providing subsidized fruit and vegetables (United Kingdom, France, and Norway), providing vegetables, fruits, and milk in schools (EU), and increasing access to fresh competitively priced fruit in hospital settings (United Kingdom). These public measures are of utmost importance in the promotion of healthy aging and could form the basis for the development of more specific guidelines regarding the implementation of broccoli sprout consumption in public settings, such as school canteens and public hospitals.

## 10. Conclusions

Envisioning the gradual aging of the population over the next few decades, it becomes crucial to discuss and bring to light new preventive approaches that promote a healthy aged population. Nutraceutical approaches, driven by food and nutrition education as well as supplementation with SFN as one of the strategies, seem to have promise for the prevention of metabolic- and immune-mediated diseases, targeting the imbalance in the redox status marked in aged individuals. Here, we discussed several beneficial effects of SFN supplementation in diseases with increased incidence in the aging population, and we proposed measures to implement nutraceuticals in this specific segment of the population.

## Figures and Tables

**Table 1:** Published in vivo interventions using either engineered mouse models or sulforaphane (SFN) administration in the context of disease.

Mouse Model Condition/Disease	Intervention	Investigated Outcomes	References
Alzheimer’s	Nrf2^−/−^	Increased beta-amyloid plaque formation	[42,43,44]
Experimental Autoimmune Encephalomyelitis	Nrf2^−/−^	Increased susceptibility to disease	[46,47]
Parkinson	Nrf2^−/−^	Increased severity of disease	[48,49]
Huntington’s disease	Nrf2^−/−^	Increased susceptibility to disease	[50]
Ischemic stroke	Nrf2^−/−^	Enhanced inflammation and cognitive impairment	[52]
Type 2 diabetes (T2D)	Nrf2-specific adipocyte KO	Decreased glucose tolerance	[60,61]
Type 2 diabetes (T2D)	Nrf2-specific hepatocyte KO	Improved insulin sensitivity	[60,61]
Autoimmunity	Keap1 −/− (systemic activation of Nrf2)	Amelioration of tissue inflammation	[65,66]
Colitis	Nrf2^−/−^	Increased severity of colitis disease	[67]
Systemic Lupus Erythematosus	Aging Nrf2^−/−^	Spontaneous development of glomerulonephritis	[68]
Cardiac and muscular dysfunction	Food SFN supplementation (442.5 mg/kg diet 2 months)	Protected mitochondrial function	[18]
Thromboembolism	Food SFN supplementation (0.125 and 0.25 mg/kg)	Inhibition of PI3K/Akt	[88]
Metabolic syndrome	Food SFN supplementation (100 µmol/kg, 6 weeks) in HFD mice	Increased insulin receptor substrate 1 (IRS-1) and GLUT4 translocations to the muscle cell membrane	[97]
Type 2 diabetes	Food SFN supplementation (2 and 10 mg/kg for 8 weeks)	Increased serum insulin levels and HOMA-β index, decreased fasting glucose	[98]
Multiple sclerosis	Food SFN supplementation (0.003%) for 18 days	Inhibition of Il23a/Il12b expression and Th17/Th1 development	[106]
Lupus	Intraperitoneal administration SFN 8 mg/kg/day, 6 weeks	Lower levels of IL-6, TNF, and IFNγ and oxidative stress	[107]
Inflammatory bowel disease	Intragastric administration SFN (20 mg/kg/day, 2 weeks)	Increased body weight, colon length, and reduced disease activity index	[112,113,114]
Bladder cancer	Food SFN supplementation (2.5 and 10 mg/kg/day 12 weeks)	Fewer tumors developed and enhanced microbiota regulation	[115]
Aging	Food SFN supplementation (442.5 mg/kg diet, 2 months)	Promotion of a younger-like microbiota	[120]
Aging	Food SFN supplementation (442.5 mg/kg diet, 2 months)	Decreased dermal layer thickness and improved collagen deposition	[73]

Abbreviations: Nrf2—erythroid 2-related factor 2; KO—knock-out; Keap1—Kelch-like ECH-associated protein 1; SFN—Sulforaphane; PI3K—Phosphoinositide 3-kinase; Akt—Ser/Thr kinase also known as Protein kinase B; HFD—High fat diet; IRS-1—Insulin receptor substrate 1; GLUT4—Glucose transporter 4; HOMA-β—homeostasis model assessment of β-cell function; Il—Interleukin; Th—T helper cells; TNF—Tumor necrosis factor; IFNγ—Interferon-gama.

**Table 2:** Published clinical trials investigating sulforaphane (SFN), broccoli, or broccoli sprouts in relation to metabolic diseases.

First Author, Year Published	Reference	Intervention (Doses/Duration)	Participants (*n*)	Study Design	Investigated Outcomes	Conclusions
Lopez-Chillon et al. (2019)	[103]	Broccoli sprouts (BS) (30 g/day) in overweight subjects, 10 wk (week) treatment, 10 wk washout	*n* = 40 healthy overweight subjects	Paired cross-over trial	IL6, CRP, TNFα, IL-1β levels	Decrease in IL6 and CRP by BS
Kikuchi et al. (2015)	[138]	3 BS extract capsules containing 30 mg of glucoraphanin/day for two months	*n* = 24 treated, *n* = 28 placebo, male subjects with fatty liver	Randomized, placebo-controlled, double-blind	Liver function markers (ALAT, ASAT, GGT, urinary 8-OHdG	Decrease in ALAT, GGT, 8-OHdG
Armah et al. (2015)	[140]	400 g broccoli (standard) or 400 g broccoli (high glucoraphanin) per wk for 12 wk treatment	*n* = 63 high glucoraphanin, *n* = 67 standard healthy volunteers	Two independent double-blind, randomly allocated parallel dietary interventions	Plasma LDL cholesterol (LDL-C) levels in middle-aged subjects	High glucoraphanin broccoli consumption decreased LDL-C
Bahadoran et al. (2012)	[95]	BS extract (high-sulforaphane), 5 or 10 g/day for 4 wk treatment	*n* = 56 treated (27 = 10 g; 29 = 5 g), *n* = 25 placebo, type 2 diabetic patients	Randomized, placebo-controlled, double-blind	Fasting serum glucose and insulin, glucose-to-insulin ratio, HOMA-IR index	10 g/d decreased fasting serum insulin and HOMA-IR
Bahadoran et al. (2011)	[139]	BS extract in tablets, 5 or 10 g/day for 4 wk treatment	*n* = 56 treated (27 = 10 g; 29 = 5 g), *n* = 25 placebo, type 2 diabetic patients	Randomized, placebo-controlled, double-blind	Serum total antioxidant capacity (TAC), total oxidant status (TOS), oxidative stress index (OSI), malondialdehyde (MDA), oxidized LDL cholesterol	Decrease in OSI, MDA, TAC, and LDL cholesterol
Christiansen et al. (2010)	[141]	Dried BS 10 g/day for 4 wk treatment	*n* = 20 treated, *n* = 20 control hypertensive individuals without diabetes (normal levels of cholesterol)	Randomized trial	Flow-mediated dilation (FMD), blood pressure, cholesterol levels	No change in FMD, blood pressure, or cholesterol levels

Abbreviations: BS—Broccoli sprouts; Il—Interleukin; CRP—C reactive protein; TNF—Tumor necrosis factor; ALAT -Alanine transaminase; ASAT—Aspartate aminotransferase; GGT—Gamma-glutamyl transferase; 8-OHdG—8-hydroxy-2′-deoxyguanosine; LDL—Low density lipoprotein; HOMA-IR—homeostasis model assessment of insulin resistance; TAC—total antioxidant capacity; TOS—total oxidant status; OSI—oxidative stress index; MDA—malondialdehyde; FMD—Flow-mediated dilation.

**Table 3:** Ongoing/unpublished clinical trials investigating sulforaphane or broccoli in heart and metabolic diseases.

Clinicaltrials.gov Identifier	Title	Institution	Intervention (Doses/Duration)	Inclusion (*n*)	Investigated Outcomes
NCT04364360 (2020)	Sulforaphane Supplementation Study (FAMOUS)	Oxford University, United Kingdom	Sulforaphane glucosinolate capsule (30 mg)/day for 3 weeks	*n* = 60 healthy adults, male or female, 18–65 years	Change in whole-body fatty acid oxidation, liver fat content, hepatic fatty acid partitioning, and glycemic control
NCT04298970 (2020)	Novel Organic Kale Products for Prevention of Obesity/Type 2 Diabetes	Aarhus University, Denmark	35–40 g of freeze-dried kale/day, NDI *	*n* = 34 type 2 diabetes, male or female, 30–75 years	The blood glucose incremental area under the curve, OGTT
NCT03763240 (2018)	Broccoli sprout extract (BSE) on Blood Glucose	Gothenburg University, Sweden	~260 mg sulforaphane/day (dry mixtures, sealed) for 12 weeks	*n* = 100 pre-diabetic individuals, 35–75 years	Fasting blood glucose, glycated hemoglobin, HOMA-IR, HOMA-B, BMI **, total cholesterol, LDL-C, HDL-C, triglycerides, fatty liver index, insulin clearance
NCT00252018 (2006)	The Effect of Broccoli Sprouts as a Nutritional Supplement in the Prevention of Cardiovascular Disease	Bispebjerg Hospital, Denmark	10 g of dried broccoli sprouts/day for four weeks	*n* = 120 in 4 groups: hypertension, diabetes, hypercholesterolemia, and healthy patients, 18–80 years	Change in endothelial function (flow-mediated dilatation (FMD))
NCT03934905 (2022)	Phase II trial of effects of the Nutritional Supplement Sulforaphane on Doxorubicin-Associated Cardiac Dysfunction	Texas Tech University Health Sciences Center and University Medical Center Lubbok	Sulforaphane caplets, daily dose, 60–240 mg for 12 weeks	*n* = 70 DOX-naïve women with breast cancer, 18–89 years	Change in cardiac function; elevation of troponin levels and tumor size
NCT05408559 (2022)	Prevention of Age-associated Cardiac and Vascular Dysfunction Using Avmacol ES (CardiacAging)	Texas Tech University Health Sciences Center	Caplets containing SFN-rich broccoli sprout extracts, daily dose for 24 weeks based on weight: two caplets for individuals <100 lb.; three caplets, 100–200 lb.; and four caplets, >200 lb.	*n* = 200, 100 men and 100 women, ≥60 years, with heart failure and preserved ejection fraction	Cardiac function, functional capacity, muscle function, hand grip test, DNA damage, oxidative stress

* NDI—no duration information; ** BMI—body mass index; OGTT—Oral glucose tolerance test; HOMA-IR—homeostasis model assessment of insulin resistance; HOMA-β—homeostasis model assessment of β-cell function; LDL-C—Low density lipoprotein Cholesterol, HDL-C—High density lipoprotein Cholesterol; FMD—flow-mediated dilatation; DOX—Doxorubicin; DNA—Deoxyribonucleic acid.

## Data Availability

This study is a narrative review of the literature. All data supporting the presentation and discussion are derived from publicly available sources cited in the article.
